# *In-vitro* evaluation of immunomodulatory activity of sulphation-modified total ginsenosides derivative-3

**DOI:** 10.3389/fvets.2023.1068315

**Published:** 2023-01-25

**Authors:** Zhiting Guo, Ling Wang, Shahbaz Ul Haq, Lu Wang, Wenzhu Guo, Yongjiang Luo, Nabeel Ijaz

**Affiliations:** ^1^Key Laboratory of New Animal Drug Project, Lanzhou, Gansu, China; ^2^Key Laboratory of Veterinary Pharmaceutical Development, Ministry of Agriculture and Rural Affairs, Lanzhou Institute of Husbandry and Pharmaceutical Sciences, Chinese Academy of Agriculture Sciences, Lanzhou, Gansu, China; ^3^Engineering Research Center of Ministry of Education for the Development and Utilization of Southwest Characteristic Medicine Biological Resources, School of Pharmacy, Guizhou University, Guiyang, China; ^4^Department of Clinical Sciences, Faculty of Veterinary Sciences, Bahauddin Zakariya University, Multan, Pakistan

**Keywords:** total ginsenosides (TG), sulphation derivative, immunological activity, antitumor, cytotoxicity

## Abstract

**Background:**

Ginseng has been used in biomedicine to prevent and treat decreased physical and mental capacities. Total ginsenosides (TG) from ginseng root which have antitumor and immune-enhancing properties, are the principal active components of *Panax ginseng*, while the sulphation-modified TG derivative-3 (SMTG-d3) was expected to enhance the anticancer activity in conventional medicinal treatments.

**Methods:**

The chlorosulphonic acid–pyridine technique, used for the sulfation modification of TG to improve their biological activity, and the infrared spectroscopic characteristics of TG and SMTG-d3 were investigated, and the effects of SMTG-d3 on immunocytes and cytokines relevant to tumor treatment were assessed. The MTT assay was used to assess the effect of TG and SMTG-d3 on the cytotoxicity and T-lymphocytic proliferation against mouse splenocytes. The LDH method was employed to evaluate NK activity induced by TG or SMTG-d3. The production levels of splenocytes-secreted IL-2 and IFN-γ and peritoneal macrophages-secreted TNF-α were determined using mouse ELISA kits.

**Results and discussion:**

It showed that the ideal conditions for the sulfation modification of TG: the volume ratio of chlorosulfonic acid to pyridine lower than 1:2.5; controlled amount of chlorosulfonic acid; and a yield of 51.5% SMTG-d3 (2 h, < 45°C). SMTG-d3 showed two characteristic absorption peaks at 1,230 cm^−1^ and 810 cm^−1^, indicating the formation of sulfuric acid esters and the presence of sulfuric acid groups. SMTG-d3 exhibited higher antitumor immunological activity than TG by promoting the proliferation of T lymphocytes and the production of IFN-γ and TNF-α, thus enhancing NK cell activity, and reducing cytotoxicity. The findings imply sulfated modification represents an effective method of enhancing the immunomodulatory activities of TG and could be used as the basis for developing new drug target compounds; SMTG-d3 can serve as an antitumor immunomodulator and can be considered an effective and prospective herbal formulation in clinical applications.

## Introduction

The field of complementary/alternative medicine is taking new horizons in the recent era. Herbal medicines are considered to be good alternatives to allopatheic medicine (1, 2). Ginseng (genus Panax, family Araliaceae, Angiosperms, accepted scientific name: *Panax ginseng* C.A. Mey.) is a medicinal herb and an agricultural product that has widely been used in many traditional medicinal therapies in China, Korea, and Japan for thousands of years. Specifically, it has been used for prophylaxis and treatment of decreased physical and mental capacities, including tiredness, exhaustion, and weakness and during convalescence (3–5). Total ginsenosides (TG), dammarane-type triterpene saponins, are the main active components in the root of *P. ginseng*. The quality and composition of the active components depend on various factors, including plant species, cultivation method, age, and the section of the plant used (6, 7). It has been shown that ginseng can inhibit the proliferation and motility of cancer cell lines *in vitro*, and that it has anticarcinogenic properties in animal models; the anticarcinogenic effects of ginseng may in part be related to its ability to inhibit angiogenesis (8–11). Furthermore, anticancer effects of TG are thought to be mediated by an increase in the production of interferons and cytokines, which can activate natural killer (NK) cells and cytotoxic T cells, thereby causing lysis of tumor cells or inhibition of tumor development (12, 13). Notably, modulation of the immune response has been recognized as the pharmacological effect of ginseng, proving that it can modulate multiple immune cell types in terms of proliferation, phagocytic activity, cytokine expression, and antibody production (14–16). Studies performed on animal models have demonstrated that ginseng stimulates a significant immune response, protecting from both viral and bacterial infections, and enhances the protection conferred by vaccine treatment (17–19).

TG is composed of sapogenin and sugar chains. Because the aglycone is a relatively stable cycloalkane structure, it is hard to modify it for structural modification of the TG molecule, whereas sugar plays an essential role in the structure–activity relationship of TG, and its modification is more purposeful. Thus, the modification of TG in this study first focused on the sulfation modification of sugar or sugar chain. Additionally, according to Miyamoto et al. (20) there are sulfate groups on the sugar chain of sea *Cucumaria ehinata* saponins, and the anticancer activity of sea cucumber saponins is related to the sulfate groups. Because the structure of sea cucumber saponins is so similar to that of TG, we believed that modification of TG by sulfation would alter their biological activity. However, it is uncertain whether the sulphation modification of TG can enhance their immunological activity even further. Thus, increasing attention has been placed on the molecular modification and structure–activity relationship of TG. The current study focused on the chlorosulphonic acid–pyridine technique for the sulfation modification of TG to improve their biological activity; and the infrared spectroscopic characteristics of TG and SMTG-d3; to investigate the effects of SMTG-d3 on immunocytes and cytokines relevant to tumor treatment were assessed. In this study, the effects of TG and sulphation-modified TG derivative-3 (SMTG-d3) on immune cells and cytokines relevant to tumor therapy were examined using mouse peripheral blood lymphocytes and mouse peritoneal macrophages cultured *in vitro*; and the alterations in immune activity for the sulfation-modified TG were assessed. This study can serve as a foundation for subsequent chemical modification, such as sulfation, to change the pharmacological activity of TG and monomers contained therein, resulting in saponin derivatives with higher biological activity and reduced toxicity.

## Materials and methods

### Chemicals and reagents

Total ginsenosides (TG) (purity: 95.60 %) were purchased from Hongjiu Biotech. Co., Ltd of Jilin Province (Changchun, China). Lipopolysaccharide (LPS), Concanavalin A (ConA), lactate dehydrogenase (LDH), and methylthiazol tetrazolium (MTT) were obtained from Sigma-Aldrich Co. (St. Louis, USA). Trypsin was obtained from Life Technologies, Inc. (Carlsbad, USA), and RPMI 1640 media and calf serum were from Gibco Co., (Grand Island, USA). Chlorosulphonic acid, pyridine, and other reagents were of analytical grade and were purchased from Tianjin Bodi Chemical Co., Ltd., (China). ELISA Kits for mouse IL-2, IFN-γ, and TNF-α were obtained from Wuhan Boster Bio. Tech., Ltd (Wuhan, China). YAC-1 mouse lymphoma cells (YAC-1 cells) were obtained from Shanghai Institute of Material Medica, CAS, China. All cells in this study were incubated in RPMI-1640 media containing calf serum (10%, v/v) and antibiotics (1%, v/v, penicillin + streptomycin), at 37°C in a humid atmosphere of 5% CO_2_. For testing, 0.25% trypsin was used to collect adherent cells in the logarithmic growth phase, and a hemocytometer was used to count the cells. For *in vitro* cytotoxicity studies, cells were seeded into new culture dishes and grown to 80 % confluence before drug treatment.

### Animals

Forty female BALB/c mice (18–22 g) with a clean grade (Certificate No. SCXK [G] 2015-001) were collected from Laboratory Animal Center, Lanzhou Veterinary Research Institute, CAAS (China). An ordinary housing facility was used and complied with the national standard, Laboratory Animal Requirements of Environment and Housing Facilities (GB 14925-2001). All animals were kept under standard environmental conditions (23–25°C; relative humidity: 50%; light/dark cycle: 12/12 h). All mice had one-week pre-experimental acclimation period and access to standard food and water ad libitum.

### Sulphation modification of TG and infrared spectroscopy analysis

Sulphation-modified TG (SMTG) was prepared using the chlorosulphonic acid–pyridine method (21, 22). In the present study, according to the volume ratio of chlorosulfonic acid to pyridine, reaction temperature, and reaction time, nine treatment schemes were designed by three-factor and three-level orthogonal test method. Nine derivatives were obtained, and nine different conditions were named in proper sequence from 1 to 9. Explicitly, TG and sulphation-modified TG derivative-3 (SMTG-d3) were used in the tests below. The yield of derivative was used as the investigation index. Yield (%) was calculated as follows: weight of products / weight of total ginsenosides × 100%.

Fourier-transform infrared (FT-IR) spectra were obtained *via* KBr tablets on a Nicolet Avatar FT-IR 360 and Nicolet FT-IR 170 SX infrared spectrophotometer (Thermo Fisher Scientific).

### T-lymphocyte proliferation test

Cytotoxicity of TG and SMTG-d3 against mouse splenocytes was evaluated by conventional MTT assay as described previously (23, 24). A suspension of mouse splenocytes was resuspended at 2.0×10^6^/mL with RPMI-1640 complete media. The cells were inoculated into 96-well plates with 100 μL per well, and treated with different concentrations of ConA-containing TG or SMTG-d3 (50 μL/well, at 5, 10, and 50 μg/mL concentrations). The final concentration of ConA in each well was 10 μg/mL. RPMI-1640 medium and ConA were applied to four wells, each for the negative control and ConA control; each sample had four replicate wells. The plates were incubated for 44 h at 37°C under 5% CO_2_. We added 20 μL of MTT per well (5.0 mg/mL) and incubated for an additional 4 h at 37°C. Then, the supernatant was aspirated, and 100 μL/well of DMSO (dimethyl sulfoxide) was added to dissolve formazan crystals existing in the viable cells. The absorbance was determined as the index of splenic T-lymphocyte proliferation using a microplate reader at 570 nm (Thermo Multiskan Mk3, USA). In addition, the cytotoxic concentrations of the drug (TG or SMTG-d3 without ConA), were determined by observing the half-inhibitory concentration of the drug on mouse T lymphocytes, and the method was the same as above.

### NK-cell activity test

YAC-1 cells were prepared at 1.0 × 10^6^/mL, and NK activity induced by TG or SMTG-d3 was measured by the LDH approach (25). Namely, 100 μL of splenocytes treated with TG or SMTG-d3 (5, 10, and 50 μg/mL) was placed in 96-well plates (20 μL drugs + 80 μL splenocytes), and then 100 μL of YAC-1 cells was added (E/T = 50/1). Additionally, the target cell's spontaneous-release wells (100 μL YAC-1 cells + 100 μL RPMI-1640) and maximum release wells (100 μL YAC-1 cells + 1% NP-40) were set as controls; each sample had four replicate wells. The plates were incubated for 24 h at 37°C under 5 % CO_2_. Then, supernatant (100 μL) was aspirated from each well, added to another culture plate, and pre-warmed at 37°C for 10 min. We then added 100 μL of LDH substrate solution freshly prepared into each well, allowed reaction at room temperature for 10–15 min (protected from light), and added 30 μL of citric acid stop solution (1.0 mol/L) into each well to stop the reaction. The absorbance was recorded using a microplate reader (570 nm).

The NK-cell activity, expressed as the percentage of release, was determined by the following formula based on the average values from the four wells: NK cells activity (%) = (values of experimental group – values of spontaneous-release group) / (values of maximum-release group – values of spontaneous-release group) × 100%.

### Detection of levels of IL-2, IFN-γ, and TNF-α secretion

In axenic conditions, splenocyte suspensions (2.0×10^6^/mL) and peritoneal macrophage suspensions (1.0×10^6^/mL) from the mice were prepared and added to 24-well plates (850 μL/well); meanwhile, 50 μL/well of ConA (in which the final concentration was 5 μg/mL) or LPS (in which the final concentration was 10 μg/mL) were added; then, TG or SMTG-d3 (at 5, 10, and 50 μg/mL concentrations) were added (100 μL/well). The plates were incubated for 24 h at 37°C in 5% CO_2_. Then, the contents of the wells were centrifuged, and the supernatants were collected (26, 27). The production levels of splenocytes-secreted IL-2 and IFN-γ, and peritoneal macrophages-secreted TNF-α, were detected using mouse ELISA kits based on the manufacturer's test instructions.

### Statistics

The data analysis was performed using SPSS Statistics 25.0 (IBM, Chicago, USA). One-way analysis of variance (ANOVA) followed by *post hoc* multiple-comparison tests (Tukey, Duncan, and Dunnett two-sided), and two-tailed *t* test (Dunnett's T3) were performed to estimate the significance of differences between the groups of control and TG or SMTG-d3. Results were expressed as mean values and standard deviations (mean ± SD), and graphs were drawn using OriginPro 2017C 64-bit (OriginLab Corporation Northampton, MA, USA). In all cases, differences were considered statistically significant when *P* was lower than 0.05.

## Results

### Sulphation modification of TG and infrared spectroscopy

According to the yield index and the degree of substitution of sulfuric acid groups, the results showed that the order of the three factors acting on the sulfation modification of TG was as follows: the temperature of the esterification reaction, the ratio of chlorosulfonic acid to pyridine, and the reaction time. However, none of the three factors significantly affected the yield index. The ideal conditions for the sulfation modification of TG were as follows: the volume ratio of chlorosulfonic acid to pyridine lower than 1:2.5; controlled amount of chlorosulfonic acid; the reaction time of 2 h; the reaction temperature lower than 45°C; and the yield of SMTG-d3 reaching 51.5 %.

**Figure 1:**
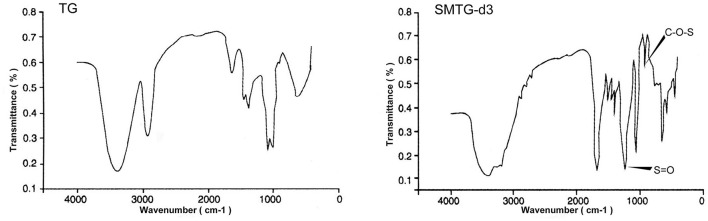
Fourier-transform infrared (FT-IR) spectroscopic analysis of TG and SMTG-d3 TG, total ginsenosides; SMTG-d3, sulphation-modified total ginsenosides derivative-3.

The FT-IR spectroscopy of TG and SMTG-d3 is shown in Figure 1. Six absorption bands at 1,638 cm^−1^, 1,330 cm^−1^, 1,254 cm^−1^, 1,037 cm^−1^, 921 cm^−1^, and 851 cm^−1^ were the characteristic absorption bands of TG; there was a strong absorption peak at 3,400 cm^−1^, indicating the existence of -OH groups; and the strong absorption at 935–1,044 cm^−1^, which indicated the stretching vibration of C-O-C (sugar ring). Compared with the IR spectrum of TG, two new characteristic absorption bands appeared in the IR spectra of SMTG-d3, one at near 1,230 cm^−1^ (describing an asymmetrical S=O stretching vibration) and the other at near 810 cm^−1^ (representing a symmetrical C-O-S vibration associated with C-O-SO_3_ group), which were the characteristic absorption peaks of sulfuric acid bonds.

### T-lymphocyte proliferation and NK-cell activity

**Figure 2:**
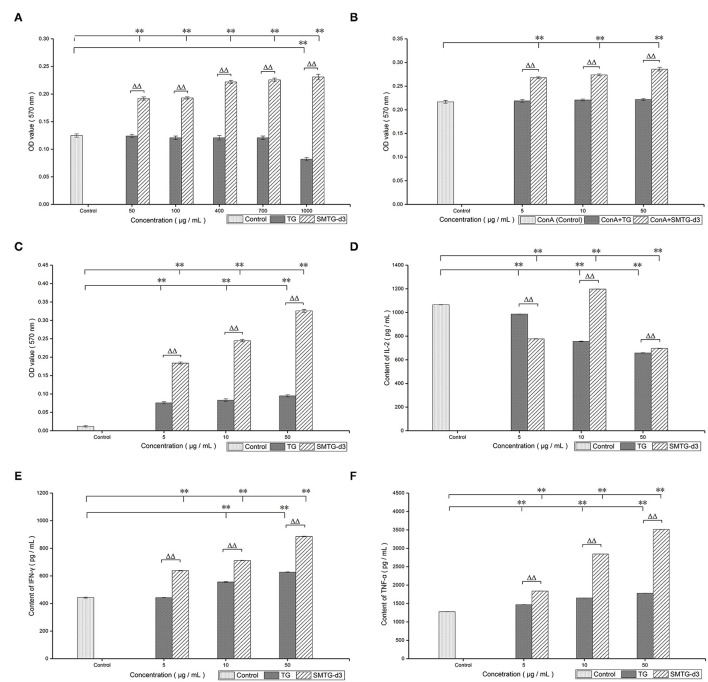
*In vitro* immunological activity of TG and SMTG-d3. Data are presented as mean ± SD. **(A)** Assay of cytotoxic concentration; **(B)** T-lymphocyte proliferation; **(C)** NK-cell activity; **(D)** IL-2 production; **(E)** IFN-γ production; **(F)** TNF-α production. *P < 0.05, **P < 0.01, compared with the control group. ^Δ^*P* < 0.05, ^ΔΔ^*P* < 0.01, compared with the TG group of the same concentration. TG, total ginsenosides; SMTG-d3, sulphation-modified total ginsenosides derivative-3.

The cytotoxicity assay of TG and SMTG-d3 on mouse T lymphocytes showed that TG significantly inhibited the proliferation of T lymphocytes at a concentration of 1,000 μg/mL (P **<** 0.01). In contrast, SMTG-d3 still had a strong promoting effect on the proliferation of T lymphocytes at the same concentration, and it significantly boosted the proliferation of splenocytes in the 50–1,000 μg/mL range (P **<** 0.01). Our findings demonstrated a considerable improvement in SMTG-d3 synergized with ConA to boost T-lymphocyte proliferation (Figures 2A, B).

Compared with the spontaneous-release group, both TG and SMTG-d3 significantly promoted the killing effect of NK cells on YAC-1 cells within the 5–50 μg/mL range (*P* **<** 0.01). Compared with the same concentration of TG, SMTG-d3 displayed more powerful capability (*P* **<** 0.01) (Figure 2C).

### Levels of IL-2, IFN-γ, and TNF-α

According to the detection of the levels of cytokines, TG exhibited an inhibitory effect on the level of IL-2 secreted by mouse T lymphocytes in the 5–50 μg/mL range after co-culture with T lymphocytes for 24 h (*P* **<** 0.01). SMTG-d3 also showed a significant inhibitory effect at the concentration of 5 μg/mL and 50 μg/mL (*P* **<** 0.01), but at the concentration of 10 μg/mL, it demonstrated a promoting effect on the production of IL-2 (*P* **<** 0.01), that is, the effect of SMTG-d3 on IL-2 secretion was of bell-jar type. Moreover, the effects of TG and SMTG-d3 on IL-2 secretion at the same concentration also showed significant differences (*P* < 0.01) (Figure 2D). After TG and SMTG-d3 were co-cultured with T lymphocytes for 24 h, respectively, the levels of IFN-γ in the culture supernatant were measured. The results indicated that both TG and SMTG-d3 were able to significantly promote the secretion of IFN-γ by mouse T lymphocytes in the range from 5 μg/mL to 50 μg/mL (*P* **<** 0.01). In general, the enhancing capacity of SMTG-d3 on IFN-γ production was significantly better than that of TG at the same concentration (*P* **<** 0.01) (Figure 2E). After TG or SMTG-d3 were co-cultured with mouse peritoneal macrophages for 24 h, TNF-α secretion in the culture supernatant exhibited higher levels compared with the control (*P* **<** 0.01); and the SMTG-d3 promoted the secretion of TNF-α significantly better than TG at the same concentration (*P* **<** 0.01) (Figure 2F).

## Discussion

The chemical modification research of natural active ingredients has now become a hot topic. Starting with the active ingredients of natural medicines, structural modification and systematic drug activity are studied to summarize the correlation between structure and activity (toxicity), which is then used as the basis for designing new drug target compounds (28–32). In a previous study, the sugar chain of TG was modified by chemical modification, and Marek's disease was employed as an animal model for the occurrence and development of viral tumors (33–36). The findings of immunological activity comparison revealed that there were fewer positive cells in the TG and its derivative drug groups than in the moroxydine group in chickens Marek's disease virus-infected; moreover, TG induced the apoptosis of MSB-1 cells (Marek's disease tumor cells) and inhibited the proliferation of MSB-1 cells. We noticed that sulfated polysaccharides often had better anti-virus, anti-tumor, and immune-improving activities (37–40), which suggests that the sulfation modification of TG should be investigated. TG are best known for their curative properties in cancer, neurodegenerative disorders, and cardiovascular diseases, and is composed of sapogenins and sugar chains. According to the structure–activity relationship of TG, their biological activity is related to the number of sugar chains present, implying that sugar chains play an important role in TG (41, 42). Thus, it is critical to sulfate the sugar chain of TG, change and enhance the biological activity, and discover more active medications from it.

In the present study, the chlorosulfonic acid–pyridine method was used to prepare sulfation-modified TG (SMTG) (21). Three main factors that affect the sulfation of polysaccharides were selected to facilitate the investigation and avoid too many experiments; these factors were the volume ratio of chlorosulfonic acid to pyridine, the reaction temperature, and the reaction time. We showed that the main factors affecting the sulfation degree of TG were the ratio of esterification agent, reaction temperature, and reaction time. We set the three levels each, and analyzed them according to the yield index through orthogonal design experiments. The results showed that the volume ratio of chlorosulfonic acid to pyridine was lower than 1:2.5; the amount of chlorosulfonic acid was controlled; the reaction time was 2 h; the reaction temperature was lower than 45°C; and the sulfation modification of TG was better. On this basis, the yield of preparing SMTG-d3 was >50%, and the effect was ideal.

The FT-IR spectrum was used to analyze the possible functional groups and bond types. Experimental results from the IR spectroscopy of TG showed that the band in the region of 3,400 cm^−1^ corresponded to the hydroxyl stretching vibration and indicated the existence of -OH groups; the band in the region of 2,920 cm^−1^ corresponded to a C-H stretching vibration; and the weak absorption bands at 1,460 cm^−1^ and 1,380 cm^−1^ corresponded to a -CH3 stretching vibration. The absorption at 1,640 cm^−1^ indicated the C=C stretching vibration; that at 1,150 cm^−1^ indicated the C-O stretching vibration of C-O-C group; and that at 1,110 cm^−1^ indicated the O-H angular vibration, which illustrated the structure of TG. In addition to retaining the characteristic absorption peaks of TG, SMTG-d3 had absorption peaks at 1,230 cm^−1^ and 810 cm-1, corresponding to the S=O stretching vibration of ${\text{OSO}}_{3}^{-}$ group and C-O-S stretching vibration, respectively. These two absorption peaks indicated that the sulphation modification had taken place in TG, which involved the formation of sulfuric acid esters and the existence of sulfuric acid groups in the molecule (Figure 1). These results showed that it was feasible to modify TG and even saponins by sulfation.

In this study, treatment with 1,000 μg/mL TG inhibited splenocyte proliferation (*P* **<** 0.01). However, SMTG-d3 significantly boosted the proliferation of splenocytes in the 50–1,000 μg / mL range (*P* **<** 0.01) (Figure 2A). These findings indicated that the sulphation modification markedly reduced the cytotoxicity of TG, and SMTG-d3 was effective for clinical practice at a lower dose. Lymphocytes, an essential component of the immune system, are involved in numerous disease-induced immunological responses. Tumor immunity is made up mostly of cellular and humoral immunity, with the former being superior to the latter (43–47). As a part of the immune response, activated T lymphocytes can secrete IFN-γ. Our findings revealed that both TG and SMTG-d3 boosted the proliferation of splenic T lymphocytes in the 5–50 μg/mL range, with SMTG-d3 producing a greater increase in T-lymphocytes proliferation than TG (*P* **<** 0.01) (Figure 2B). ConA stimulates T lymphocytes primarily as a mitogen; thus, treatment with ConA induces the cells to move into G_1_ phase from G_0_ phase (DNA presynthetic phase); otherwise, the cells remain in the G_0_ phase. In this study, the proliferation of T lymphocytes was significantly enhanced by SMTG-d3, indicating that SMTG-d3 caused a large number of T lymphocytes to transition from a resting to an active state by regulating the cell proliferation cycle. Furthermore, compared with the same concentration of TG, SMTG-d3 had an obvious synergistic effect with ConA, and showed a significant positive result in promoting T-lymphocyte proliferation *in vitro*.

NK cells can directly destroy tumor cells after activation and secrete some cytokines, such as interleukins and interferon, which participate in immune killing and immune elimination (45, 48). Our data showed that TG and SMTG-d3 both increased the NK-cell activity in the 5–50 μg / mL range (*P* **<** 0.01); however, SMTG-d3 was more effective than TG at enhancing NK cell activity (*P* **<** 0.01) (Figure 2C). These findings indicate that SMTG-d3 may effectively kill YAC-1 cells or inhibit tumor cell development. Numerous crucial physiological processes, including immune response and regulation, and stimulation of cell proliferation and differentiation *in vivo*, are affected by cytokines. To further explore the cellular immune regulation mechanism of TG and SMTG-d3, and given that both TG and SMTG-d3 had good proliferative effects on T lymphocytes *in vitro*, the effects of TG and SMTG-d3 on the cytokines released by lymphocytes and macrophages were further investigated. IL-2 is produced by activated T lymphocytes and mainly promotes the activation and proliferation of T and B lymphocytes. After T lymphocytes are stimulated by ConA, they begin to secrete IL-2; meanwhile, they also express a high level of IL-2R. We demonstrated that both TG and SMTG-d3 had obvious inhibitory effects on the secretion of IL-2 by mouse T lymphocytes within the concentration range in this test (*P* **<** 0.01) (Figure 2D). Compared with the same concentration of TG, the effect of SMTG-d3 on inhibiting the secretion of IL-2 from T lymphocytes was significantly weaker (*P* **<** 0.01) (at the concentration of 10 μg/mL). The inhibitory effect of TG was in a negative dose–response relationship, and the secretion effect of SMTG-d3 on IL-2 was related to the dosage, showing a bell-jar type. The results above could be attributed to the antagonism of ConA by TG or SMTG-d3, as well as the decreased expression of IL-2R, and suppression of the proliferation signal. In earlier research (49–52), it was discovered that ginsenosides improved the reduction of IL-2 secretion in experimental animals under immunosuppression conditions, but had no appreciable impact on the immune function in healthy animals, which might indicate the immunomodulatory effect of ginsenosides. The difference between our findings and those of the previous studies may stem from the differences between *in vivo* and *in vitro* testing, and pathology and health, and further research is necessary to determine the action mechanism.

Many studies shown that activated T lymphocytes secrete IFN-γ, which can directly inhibit the proliferation of tumor cells and boost the expression of TNF-α; TNF-α can selectively destroy tumor cells or permit not-yet-diseased cells to move into an anti-tumor condition in a paracrine manner (3, 12, 53–55). Our data showed that both TG and SMTG-d3 significantly promoted the production of IFN-γ and TNF-α within the range of 5–50 μg/mL concentration (*P* **<** 0.01); and compared with TG at the same concentration, SMTG-d3 promoted the secretion of IFN-γ and TNF-α more considerably (*P* **<** 0.01) (Figures 2E, F). Many studies have suggested that the production of IFN-γ is inhibited in the whole organism and tumor region in cancer patients (5, 12, 38, 49). Recent discoveries have revealed that TG and their derivatives play an important role in assisting tumor cells in apoptosis and differentiation, as well as in increasing tumor cell sensitivity to chemotherapeutic treatments. TG in combination with chemotherapeutic medicines are currently utilized mostly to improve the efficacy of primary lung cancer and liver cancer treatment (5, 56, 57). Thus, promoting the production of IFN-γ by SMTG-d3 may be beneficial during radiotherapy and chemotherapy. Findinds above implying that SMTG-d3 could work as an antitumor immunomodulator, and serve as foundation for subsequent chemical modification, such as sulfation to change the pharmacological activity for TG and monomers contained therein, resulting in saponin derivatives with higher biological activity and reduced toxicity.

## Conclusions

The capacity of TG and SMTG-d3 to improve immunity was discovered to be one of the primary mechanisms underlying their antitumor activity. For their immunoregulatory impact, TG and SMTG-d3 increased the activity of T lymphocytes, NK cells, and peritoneal macrophages, and aided in the production of IFN-γ and TNF-α throughout the antitumor process. Moreover, compared with TG, SMTG-d3 further enhanced antitumor immunity while lowering cytotoxicity. Hence, using ginseng's active ingredients as a starting point, and examining the relationship between structural modification and activity (toxicity), could be used as the basis for developing new drug target compounds; and the customized derivatives with desirable functional characteristics can be produced. SMTG-d3 appears to be a promising antitumor immunomodulator, and it was expected to give greater value to the anticancer activity in traditional medicinal therapies.

## Data availability statement

The original contributions presented in the study are included in the article/Supplementary material, further inquiries can be directed to the corresponding author.

## Ethics statement

All animal experiments strictly abided by the National Institutes of Health (NIH) Guidelines for Laboratory Animal Treatment and Use (58), and the study was approved by the Ethics Committee of Lanzhou Institute of Husbandry and Pharmaceutical Sciences of the Chinese Academy of Agricultural Sciences (Lanzhou, China).

## Author contributions

ZG and LiW conceived and designed the experiments, performed the experiments, analyzed the data, prepared figures, authored or reviewed drafts of the article, and approved the final draft. SH performed the experiments, analyzed the data, prepared figures, authored or reviewed drafts of the article, and approved the final draft. LuW conceived and designed the experiments, performed the experiments, analyzed the data, prepared figures, and approved the final draft. WG and YL performed the experiments, analyzed the data, authored or reviewed drafts of the article, and approved the final draft. NI helped modify the final version manuscript and approved the final draft. All authors contributed to the article and approved the submitted version.
