# Supplementary material for: In-vitro evaluation of immunomodulatory activity of sulphation-modified total ginsenosides derivative-3
Source: Front Vet Sci. 2023 Jan 25;10:1068315. doi: 10.3389/fvets.2023.1068315 (PMC9907730; doi:10.3389/fvets.2023.1068315)
Supplement: Supplementary file 1 [file Data_Sheet_1.zip › 20221009-SMTG-Supplementary material-1.doc]

**Supplementary material**

**1. Supplementary Data**

**(A) Assay of cytotoxic concentration**

|  | Control | TG | SMTG-d3 |
| --- | --- | --- | --- |
| Control | 0.125±0.003 |  |  |
| 50 |  | 0.124±0.003 | 0.192±0.003 |
| 100 |  | 0.121±0.003 | 0.193±0.002 |
| 400 |  | 0.121±0.004 | 0.222±0.003 |
| 700 |  | 0.121±0.003 | 0.223±0.003 |
| 1000 |  | 0.082±0.003 | 0.231±0.005 |

**(B) T lymphocyte proliferation**

|  | ConA(Control) | ConA+TG | ConA+SMTG-d3 |
| --- | --- | --- | --- |
| ConA(Control) | 0.217±0.003 |  |  |
| 5 |  | 0.219±0.003 | 0.268±0.002 |
| 10 |  | 0.221±0.005 | 0.272±0.002 |
| 50 |  | 0.222±0.002 | 0.286±0.004 |

**(C) NK cell activity**

|  | ConA(Control) | ConA+TG | ConA+SMTG-d3 |
| --- | --- | --- | --- |
| ConA(Control) | 0.012±0.002 |  |  |
| 5 |  | 0.076±0.003 | 0.184±0.003 |
| 10 |  | 0.083±0.004 | 0.245±0.003 |
| 50 |  | 0.095±0.003 | 0.326±0.004 |

**(D) IL-2 production**

|  | Control | TG | SMTG-d3 |
| --- | --- | --- | --- |
| Control | 1065.83±1.59 |  |  |
| 5 |  | 986.54±1.26 | 777.43±1.63 |
| 10 |  | 755.99±2.70 | 1198.52±0.96 |
| 50 |  | 657.85±2.12 | 696.39±1.52 |

**(E) IFN-γ production**

|  | Control | TG | SMTG-d3 |
| --- | --- | --- | --- |
| Control | 443.01±3.81 |  |  |
| 5 |  | 442.74±1.82 | 638.60±1.84 |
| 10 |  | 556.05±3.36 | 712.21±1.37 |
| 50 |  | 627.50±1.64 | 885.90±1.96 |

**(F) TNF-α production**

|  | Control | TG | SMTG-d3 |
| --- | --- | --- | --- |
| Control | 1276.99±1.79 |  |  |
| 5 |  | 1472.29±2.05 | 1840.71±1.26 |
| 10 |  | 1652.35±0.71 | 2846.05±1.37 |
| 50 |  | 1780.76±1.34 | 3513.78±2.30 |

**2. Supplementary Figures**

**Figure S1.** Fourier transform infrared (FT-IR) spectroscopic analysis of TG and SMTG-d3.

TG, total ginsensides; SMTG-d3, sulphation-modified total ginsenosides derivative-3

**Figure S2.** In vitro immunological activity of TG and SMTG-d3. Data are presented as mean ± SD. * P < 0.05, ** P < 0.01, compared with control group; ^∆^ P < 0.05, ^∆ ∆^ P < 0.01, compared with TG group of same concentration.

(A) Assay of cytotoxic concentration	(B) T lymphocyte proliferation	(C) NK cell activity (D) IL-2 production	(E) IFN-γ production	(F) TNF-α production

e

TG, total ginsensides; SMTG-d3, sulphation-modified total ginsenosides derivative-3.
